# Ratiometric Decoding of Pheromones for a Biomimetic Infochemical Communication System

**DOI:** 10.3390/s17112489

**Published:** 2017-10-30

**Authors:** Guangfen Wei, Sanju Thomas, Marina Cole, Zoltán Rácz, Julian W. Gardner

**Affiliations:** 1 Microsensors and Bioelectronics Laboratory, School of Engineering, University of Warwick, Coventry CV4 7AL, UK; s.thomas.1@warwick.ac.uk (S.T.); marina.cole@warwick.ac.uk (M.C.); zoltan.racz@durham.ac.uk (Z.R.); J.W.Gardner@warwick.ac.uk (J.W.G.); 2 School of Information & Electronic Engineering, Shandong Technology and Business University, Yantai 264005, China; 3 School of Engineering and Computing Sciences, Durham University, Durham DH1 3LE, UK

**Keywords:** ratiometric decoding, pheromone, biomimetic infochemical communication, VOC detection, SAW sensor array

## Abstract

Biosynthetic infochemical communication is an emerging scientific field employing molecular compounds for information transmission, labelling, and biochemical interfacing; having potential application in diverse areas ranging from pest management to group coordination of swarming robots. Our communication system comprises a chemoemitter module that encodes information by producing volatile pheromone components and a chemoreceiver module that decodes the transmitted ratiometric information via polymer-coated piezoelectric Surface Acoustic Wave Resonator (SAWR) sensors. The inspiration for such a system is based on the pheromone-based communication between insects. Ten features are extracted from the SAWR sensor response and analysed using multi-variate classification techniques, i.e., Linear Discriminant Analysis (LDA), Probabilistic Neural Network (PNN), and Multilayer Perception Neural Network (MLPNN) methods, and an optimal feature subset is identified. A combination of steady state and transient features of the sensor signals showed superior performances with LDA and MLPNN. Although MLPNN gave excellent results reaching 100% recognition rate at 400 s, over all time stations PNN gave the best performance based on an expanded data-set with adjacent neighbours. In this case, 100% of the pheromone mixtures were successfully identified just 200 s after they were first injected into the wind tunnel. We believe that this approach can be used for future chemical communication employing simple mixtures of airborne molecules.

## 1. Introduction

For over half a century, *biomimetics* has evolved as a research discipline studying biological characteristics and operational principles of living organisms to apply in areas such as robotics, sensor and actuator design, complex system integration, and artificial intelligence [1]. Many species of insects, with their sensitive olfactory system, rely on volatile pheromones as signal messengers to locate their mates, detect food sources, organize groups, and avoid dangers from predators. These ratiometric chemical messages are decoded by the peripheral olfactory system of the insect, which is subsequently translated into robust behavioural responses under turbulent, real-world conditions [2]. Insects use their antennae to detect the nature, intensity, and gradient direction of volatile chemical compounds. This form of communication using chemicals alone has promoted the development of a new class of technology for labeling, information transmission and biochemical interfacing [3]. *Biosynthetic infochemical communication* could also be applied to automated identification and data capture, product labeling, search and rescue missions, air silent communication, medical diagnosis/treatment, environmental monitoring, and source localization.

Insects, in particular moths, utilize blends of different chemical compounds to communicate with their sophisticated chemical signaling and processing systems [4]. As the species-specific blend ratio is crucial for male orientation to a female emitter [5], specificity is achieved by precise blends of pheromone components with variation in molecular chain length, unsaturation level, functional group, and total number of compounds; rather than by chemically unique structures [6]. These infochemical signal blends are produced by generating a spatially and temporally defined combination of different semio-chemicals that are often biosynthesized in the insect’s pheromone gland. Female moths release tiny quantities of pheromone blends into the air, which are detected several miles away by the extremely sensitive feathery antennal system of the male moths. Component-specific receptor proteins present in the antennal sensory neurons of the male moths are activated by these odour cues, producing electrical potentials conferring information about the odorant molecule [7]. These electrical signals are processed into a unified, ratiometric signal within the antennal lobe of the brain [8].

The biological inspiration for the work described in this paper is the moth ‘*Spodoptera littoralis*’ [9,10] because its infochemical (e.g., sex pheromones) mediated behaviour is well established, both from a behavioural and neurophysiological point of view. The biosynthetic stages of generation of these pheromones have also been well-defined [11].

An artificial system mimicking moth-pheromone communication has been developed to demonstrate pheromone-based ratiometric information encoding, transmission, and decoding. This innovative modular system consists of a *chemoemitter* that is capable of releasing a predefined ratio of volatile compounds, and a chemoreceiver that is capable of detecting the volatile compounds in air.

**Figure 1:**
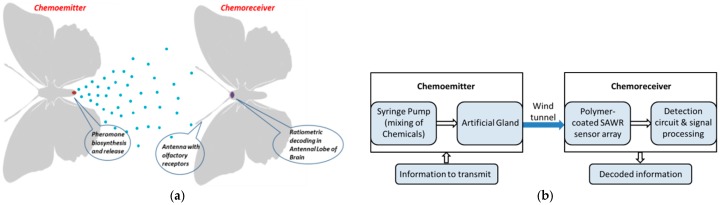
(**a**) Schematic diagram showing the relation between the primary biological components contributing to pheromone-based insect communication; and (**b**) the corresponding bio-inspired modules that form a possible configuration of a biosynthetic infochemical communication system with chemoemitter and chemoreceiver.

The chemoemitter utilizes an artificial gland [12] that mimics pheromone production and release by a female moth. The chemoreceiver consists of a polymer-coated piezoelectric Surface Acoustic Wave Resonator (SAWR) sensor array, which mimics the molecular detection in the insect antenna of the male moth. The signal processing in the antennal lobe of the insect brain is simulated by the recovery of ratiometric information contained in the odour cues by the use of a biologically constrained signal-processing model. The modular arrangement of the chemoemitter and chemoreceiver [13] sections of an infochemical communication system prototype is shown in Figure 1; it gives the relation between the biological and technological counterparts.

In this paper, a method to detect biosynthetic ligands using the SAWR sensor system is described, as well as the decoding of the ratiometric information using various neural network algorithms. As an approach for rapidly decoding ratiometric infochemical signals in real-time, the applications of Linear Discriminant Analysis (LDA), Probabilistic Neural Networks (PNN), and Multilayer Perception Neural Network (MLPNN) algorithms are explored using different feature extraction methods of SAWR sensor signals. With complementarily-tuned sensors and advanced data analysis models, the presented infochemical system demonstrates the potential for autonomous communication by mimicking the pheromone-based communication between insects.

## 2. Infochemical Communication System

**Figure 2:**
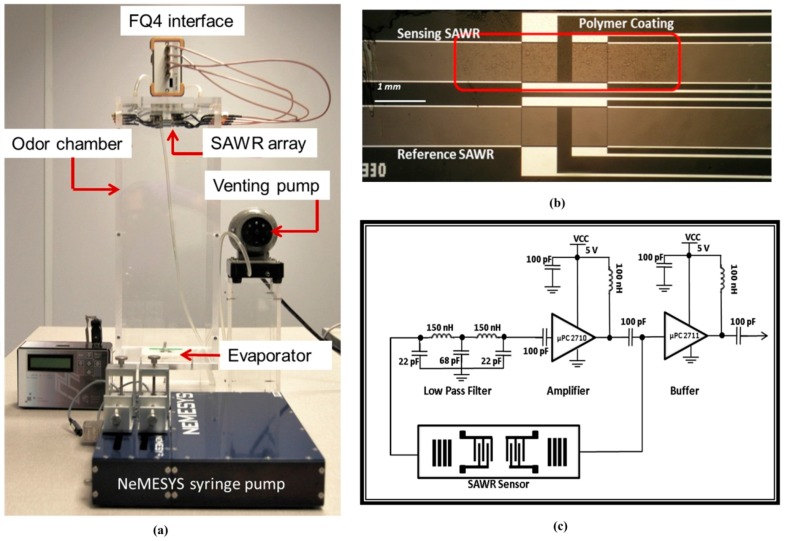
(**a**) Prototype of an infochemical test chamber containing syringe pump, micro-evaporator and array of polymer-coated SAWR sensor. (**b**) Optical micrograph of a 263 MHz Rayleigh mode dual SAWR with the polymer indicated on the sensing device. (**c**) Schematic block diagram of the feedback-loop oscillator circuit used for the pheromone-based ratiometric measurements.

The basic configuration of the communication system involves a microsystem, called the *chemoemitter module*, which is capable of producing and releasing a precise mixture of ratiometric biosynthetic pheromone compounds, and a sensor system, called the *chemoreceiver module*, which is capable of detecting these biosynthetic ligands and decoding the ratiometrically encoded information [11,14,15,16]. A prototype of the modular infochemical communication system has been constructed and assembled, as shown in Figure 2a.

The chemoemitter module comprises of a neMESYS high-precision multi-channel syringe pump (Cetoni GmbH, Korbussen, Germany) that drives a micro-machined evaporator (or “artificial gland”) releasing pheromones into the chamber. Precisely controlled dilution and mixing of the compounds is used to produce the pheromone blends with encoded ratiometric information. These blends are released into the environment as time-sensitive and time-registered information via controlled thermal volatilization using the artificial gland and its associated controller. The Perspex odour chamber (14 cm × 14 cm × 40 cm in volume), which forms the wind tunnel, is coupled to a membrane venting pump, in order to transport the odour cues to the chemoreceiver module situated at the opposite side of the chamber.

The chemoreceiver detects and recovers the ratiometric chemical information via an array of robust and highly sensitive functionalized 263 MHz SAW resonator devices. The SAWR sensors are coated with a chemically- selective functional coating (i.e., polymer) to concentrate vapour molecules on the device surface. Polyethylene (PE), polystyrene-co-butadiene (PSB), and polyethylene-co-vinyl acetate (PEVA) were selected based on their Volatile Organic Compound (VOC) sensitivity and selectivity (i.e., high partition coefficients). The polymers were airbrushed onto the sensing SAWR device surfaces to a thickness of about ~15 nm, as detailed in [11]. This was achieved by monitoring the SAWR resonant frequency shift during the polymer coating process.

Absorption of the vapour molecules into the coating changes the propagation of the acoustic wave, bringing about changes in wave velocity, which can be measured indirectly, as a shift in the resonant frequency. Figure 2b shows the optical micrograph of the polymer coated sensing side and the uncoated reference side of a 263 MHz Rayleigh mode dual SAWR sensor fabricated on ST-cut quartz substrates using Al electrodes. These sensors are designed in a dual configuration in order to ameliorate the effect of common mode interferences [17]. The SAW resonators are driven by simple feedback based oscillator circuits as outlined in Figure 2c, which provide highly stable and precise frequency measurements by detecting the SAW propagation characteristics.

## 3. Experimental Procedure

The sex pheromone of the Egyptian cotton leafworm (a noctuid moth) *Spodoptera littoralis* has been identified as a blend of several compounds including-(Z,E)-9,11-tetradecadienyl acetate ((Z,E)-9,11-14:OAc), (Z)-9-tetradecenyl acetate(*Z*9-14:OAc), (E)-11-tetradecenyl acetate (E11-14:OAc), and tetradecyl acetate (14:OAc) [9,10]. However, the female pheromone composition of this moth varies heavily depending upon the origin of the strain [9,10,18]. A summary of the sex pheromone compositions of *Spodoptera littoralis* has been reported in [18], which shows that Z9-14:OAc and E11-14:OAc forms the two major secondary components, whose ratios vary immensely depending on the origin of the strain. Hence E11-14:OAc and Z9-14:OAc were chosen as the two pheromone compounds to encode the ratiometric information, so as to validate the performance of the infochemical communication system. These pheromone compounds were biosynthesized by Dimov et al. [19] using a silicon-glass based microreactor coated with anti-adsorption polyelectrolyte multilayer. This MEMS based biosynthetic reactor forms part of an ‘‘artificial gland’’, as described in [12], and is capable of producing a pre-defined amount of the pheromone component. Details of the pheromone synthesis pathways can be found in reference [18].

Ten binary volatile pheromone blends were used as the ratiometric input dataset, as shown in Table 1. The ratios of the two pheromones were set to 1:0, 2:1, 1:1, 1:2, and 0:1, within the range of volumes of 0 to 2 μL. Concentrated pheromone compounds were utilized for the preparation of the ratios. The selection of the ratios were purely random because of the fact that the natural ratios vary heavily based on the species origin [18]. Moreover, the purpose of our experiments is to realize an effective decoding method to recover any ratiometric information successfully. This helps in the conservation of blend information between the source and the receiver. 

**Figure 3:**
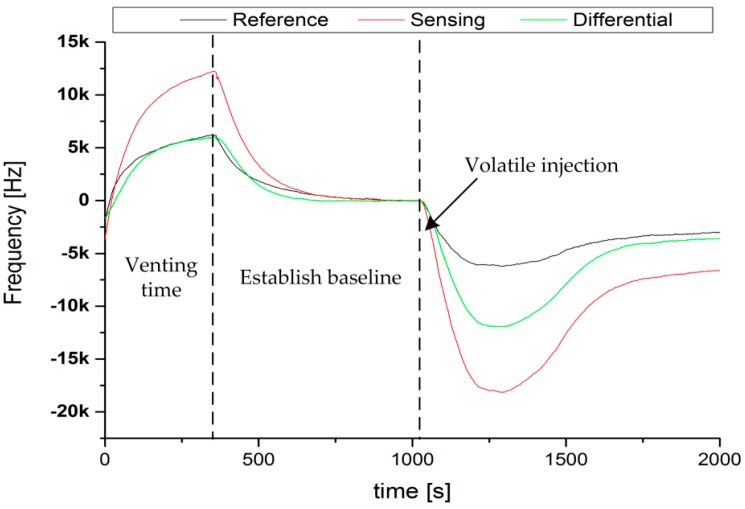
Surface Acoustic Wave Resonator (SAWR) responses obtained during a typical ratiometric measurement, (0.33 μL Z9-14:OAc and 0.66 μL E11-14:OAc) showing sensing, reference and difference signals of a PSB-coated dual sensor. The sensor response during venting and baseline establishment is also shown.

Before the start of each experiment, normal clean air was pumped into the odour chamber for the purpose of purging the chamber to remove any residual volatile vapours from previous measurements. This allowed the sensor signals to return to their initial ambient conditions. A pheromone blend was carried and injected by capillary tubes from the syringe pump to the microfluidic channels of the micro evaporator, after the baseline of the sensor signals became stable. The venting and baseline establishment times are clearly shown in Figure 3. Frequency responses of each individual SAWR of the sensor array to the pheromone ratios were carefully recorded as a time series using a commercial interface board (JLM Innovation, Tübingen, Germany).

The syringe pump based chemoemitter was utilized to inject different volume concentrations of pheromone E11-14:OAc and Z9-14:OAc into the odour chamber in randomized repeated sequences. The typical frequency response of a polymer-coated dual SAWR sensor to an injection of 1:2 ratio of pheromone blends of Z9-14:OAc and E11-14:OAc, respectively, is shown in Figure 3. As is evident from Figure 3, a frequency shift was observed in both the coated and the uncoated sensor devices, due to the addition of volatile molecules on to the sensor surface. A differential frequency response of the SAWR sensor array was obtained, which removed common mode variations and produced a frequency shift corresponding to the pheromone blend. Also, the differential signal suggests that the response of the polymer coated SAWR is much greater than the reference signal.

**Figure 4:**
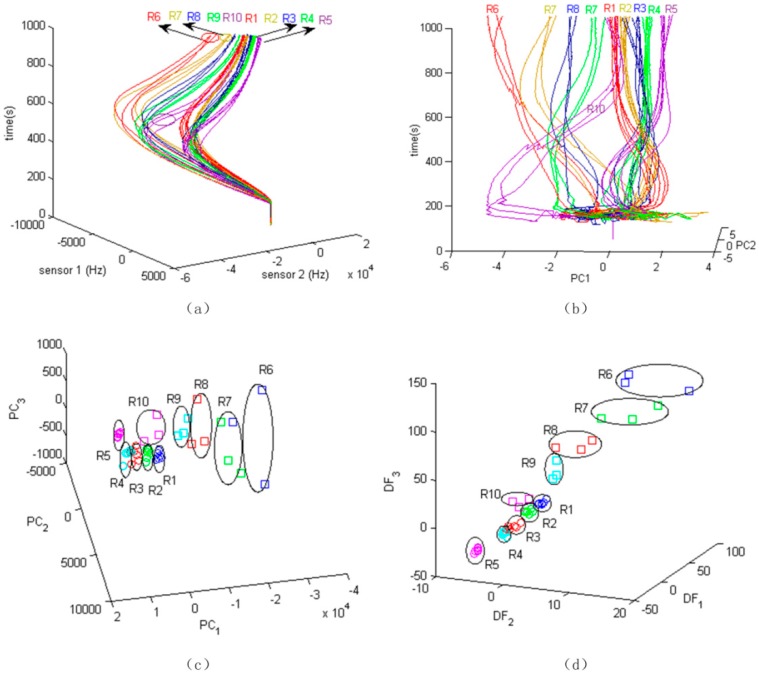
(**a**) Time development trajectories based on Sensor 1 (PE) and Sensor 2 (PSB) data; (**b**) Time development trajectories based on PC1 and PC2 scores; (**c**) three-dimensional (3D) scatter plot of Principal components; and, (**d**) discriminate functions of the pheromone blend data points at *t* = 650 s after injection.

For the low-volume 5 ratios, measurements were repeated six times and for high-volume 5 ratios, measurements were repeated three times, all randomly. Responses of the three sensors to 10 categories of all the repeats were recorded, which added up to a total of 3 (sensors) × 5 (low-volume ratios) × 6 (repeats) + 3 (sensors) × 5 (high-volume ratios) × 3 (repeats) = 3 × 45 signal profiles. The 3D plot of the time trajectory based on two sensors of the SAWR sensor array is shown in Figure 4a. It can be roughly seen that the frequency responses of high volume ratios are larger than the low volume ratios. 

### 3.1. Repeatability and Correlation of Sensor Responses

The average value and standard deviation of sensor response to each category at each time were calculated. The repeatability of the sensors information was characterized by calculating the ratio of standard deviation and the average values, which is the statistical deviation percentage or standard error of the sensor responses from the average. Based on the calculated results, the deviation percentage of each sensor is random and widely distributed when there is no injection of the pheromone ratios (because the baseline of frequency shift is around zero). After the injection of pheromone, the sensor deviation decreases. When a sensor starts to respond, the deviation level decreases rapidly and reaches a relatively stable level, which is lower than 10%. Minimum deviation occurs at the highest frequency shifts for most sensors. Good repeatability is shown by PEVA and PSB based SAWR sensors towards the steady-state level. However, the deviation level is high for PE-based SAWR sensors towards the end of the measurements, when compared to other sensor types.

The correlation between the different SAWR sensor responses was determined to quantify the selectivity of the polymer layers. The mean correlation coefficients between PE and PSB, between PSB and PEVA, between PE and PEVA based sensors are 0.963, 0.975, and 0.902, respectively, which show that the sensor responses are strongly correlated. This is to be expected when trying to separate very chemical compounds structurally similar.

### 3.2. Principal Component Analysis

Principal Component Analysis (PCA) is generally used to reduce feature dimensionality and display signal variations. Frequency shifts of the sensors were used as features in a PCA, in order to evaluate the ratiometric decoding capability of the infochemical communication system. Figure 4b shows the time dependent trajectory based on the first two principal components (PC1 and PC2). With time, the data points of each category group together. Starting from *t* = 250 s, it can be observed that high concentration ratio points are separated clearly from the low concentration ratio points. The 10 categories do show considerable intertwining. R1, R2, and R10 overlap each other clearly. The curves of each category cluster as time increases, but they are entangled from the start of the injection of pheromones to 600 s. PCA shows some group separation (in reduced multi-linear space), however the lines are still overlapping. Three-dimensional (3D) plots of the PCs at 650 s are shown in Figure 4c. Overlapping of groups (i.e., ratios) exists and it will bring more recognition errors.

In order to decode properly the signals it is clearly necessary to use more advanced feature sets and non-linear pattern recognition classifiers, and so other features are extracted and studied with the non-linear, non-parametric, data-driven methods of LDA, MLPNN, and PNN.

## 4. Feature Extraction

Features are extracted from the sensor responses in order to obtain good decoding performances, such as high rate and speed of recognition, mainly showing the effectiveness of decoding and classifying the transmitted information [20,21,22]. Table 2 lists the features selected and analyzed in this study. It has been shown elsewhere that the response of polymer-based chemical sensor can be approximated by a first-order exponential model [23]. Similarly, the frequency response of the SAWR sensors can be expressed in terms of the rise time, as shown in Equation (1):

(1) f(t)=f(∞)[1−exp(−t/τ)]

where f(∞) is the frequency shift at the stationary state and is expected to be the maximum value, and τ is the characteristic rise time. Rearranging the equation, the rise time τ is inferred as the reciprocal of derivative of the logarithm of response signal.

(2) $$\tau =-1/\left\{\frac{d\left\{\ln\left[f\left(\infty \right)-f\left(t\right)\right]\right\}}{dt}\right\}$$

If $0<\frac{t}{\tau }\;\ll 1$, the rise time τ can be also obtained by the derivative of response signal directly, as derived in Equation (3).

(3) $$\tau =\frac{1}{\left[\frac{df\left(t\right)}{dt}\frac{1}{f\left(\infty \right)}\right]}=\frac{f\left(\infty \right)}{\left[\frac{df\left(t\right)}{dt}\right]}$$

## 5. Decoding Algorithms

10 categories of pheromone mixtures at five ratios and two volumes were defined as the target biosynthetic chemical codes. For a comparative study, general pattern recognition approaches can be applied as decoding algorithms. Principal Components Analysis (PCA) [24,25] has been widely used as a group display technique in electronic noses. Fisher's linear discriminate analysis (LDA) is also a typical linear classification technique applied in electronic nose applications [20,26]. Other than these linear techniques, nonlinear techniques, including artificial neural networks (ANN) and probabilistic neural networks (PNN), are the most studied recognition methods in gas sensing area [27]. These powerful nonlinear classifiers are adopted and studied as the decoding methods in the following subsections. Performances, including recognition rate and speed, are analysed and compared.

### 5.1. LDA Based Representation and Classification

The infochemical codes (or labels) are associated with discrimination capabilities (e.g., inter-class distance) by signal classification methods. Both LDA and PCA are linear classification techniques, which are able to present the points in a transformed space and show inner-class distance and inter-class distance. Figure 4c,d show the data points at *t* = 650 s after injection of the pheromone blends in the 3D space of Principal Components (PCs), and the space of Discriminate Functions (DFs). In the PC space, the data-points are distributed based on the major variation direction of the original data. But due to the large deviation of frequency shift, groups clearly overlap with each other. Because the LDA considers the overall discrimination between groups, the groups in DF space are more centered and with larger distances between them. The classification is then performed based upon these canonical functions. Subjects are classified in the groups in which they had the best classification scores. Here, a linear function is adopted as the classifier. 

For the purpose of robust training with small sample sets, the Leave-One-Out verification method is adopted to demonstrate the process. A sample is randomly chosen as the test vector by this method and the remaining44 samples are then used as training vectors. This process is repeated for the remaining unchosen samples. Finally, the overall average recognition rate is computed for each of the different discrete sample times, e.g., *t* = 200 s.

### 5.2. Probabilistic Neural Network Based Classification

A Probabilistic Neural Network (PNN) [28,29,30] approach employs a supervised neural network that is closely related to the Bayes classification rule and the Parzen nonparametric probability density function estimation theory. PNNs offer a way to classify multi-dimensional vectors using probability density functions (often Gaussian).

It is not necessary to calculate the full probability density function when using Parzen windows for classification; it is sufficient to evaluate it at the test vector point. The following equation expresses the method for finding the needed value, extended to the *n*-dimensional case:

(4) $$f_{a}\left(X\right)=1/{\left(2\pi \right)}^{\frac{p}{2}}\sigma^{p}\left(1/n_{a}\right)\overset{n_{a}}{\underset{i=1}{\sum }}\exp\left(-\frac{{\left(X-Y_{ai}\right)}^{t}\left(X-Y_{ai}\right)}{2\sigma^{2}}\right)$$

where $f_{a}\left(X\right)$ is the value of the probability density function of class A at point ***X***, i is the number of the training vector, p is the number of components in the training vector, σ is a smoothing parameter, $n_{a}$ is the number of training vectors in class A, X is the test vector to be classified, $Y_{ai}$ is the *i*th training vector from class A, and finally, superscript t denotes the transpose.

**Figure 5:**
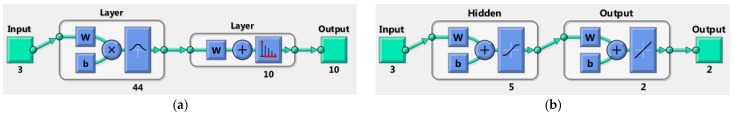
Ratiometric decoding structures of (**a**) Probabilistic Neural Network (PNN) and (**b**) Multilayer Perception Neural Network (MLPNN).

The simple PNN structure used here is shown in Figure 5a. When an input is presented, the first layer computes the distance (in multi-dimensional space) from the input vector to a training input vector and produces a vector whose elements indicate how close the input is to the desired value. The second layer sums these contributions for each class of inputs to produce as its net output a vector of probabilities. Finally, a transfer function is applied to the output of the second layer and picks the maximum of these probabilities and produces a binary “1” for that class and a “0” for other classes. The 10 ratios are encoded as numbers ranging from1 to 10. The input is the verification sample randomly generated by the Leave-One-Out method, the remaining 44 samples are the pattern layer nodes. The average classification/recognition rate of all repeated results is defined as the recognition ratio.

The smoothing parameter σ is also the standard deviation of the Gaussian, and it must be pre-selected to provide an appropriate width for the distribution. If the value chosen for σ is small, then each Gaussian is narrow; whereas, if the value of σ is made larger, the Gaussian functions are spread out and flattened, producing a smoother probability density function estimate. Here, the classification accuracy was found to be relatively insensitive to the exact value of σ. As σ approaches 0, a nearest neighbour classifier is in effect approximated. As it approaches infinity, the decision boundaries approach the hyperplanes, thus limiting the classifier to functions that are linearly separable like LDA. In this paper, as the feature values varied over a large range, a constant smoothing parameter is inappropriate unless all of the features are normalized (i.e., autoranged) at the first step. However, the normalization does affect the data structure and to keep the consistent data structure for the purpose of comparison, the data were not normalized at first, and instead a different smoothing parameter was calculated for each feature.

It is evident that the appropriate value for the spread parameter of each class depends on the distances between the adjacent samples of that class. *D_i_* is defined as the set of distances between the samples of the *i*th class and their nearest neighbours in the same class:

$$D_{i}=\left\{\min\left\{\mathrm{dist}\left(s_{k},s_{j}\right);j=1,\cdots N_{i},j\neq k,\;k=1,\cdots N_{i}\right\}\right\},\;i=1,\;\cdots c$$

where $\mathrm{dist}\left(s_{k},s_{j}\right)$ is the Euclidean (linear) distance between two sample vectors $s_{k}$ and $s_{j}$, $N_{i}$ is the number of samples in the *i*th class, ***c*** is the number of classes. Several ways were examined for obtaining an appropriate value for spread parameter from *D_i_,* including the smallest member of *D_i_*, average of *D_i_* members, median of *D_i_* members, the largest member of *D_i_*. It was found that usually the $median(D_{i})$ is the most appropriate estimate for spread parameter.

### 5.3. Back-Propagation Multilayer Neural Network Based Classification

The Rumelhart back-propagation (BP) multilayer neural network is a widely used pattern recognition approach in odour classification research. For comparison, the BP-based MLPNN is adopted as the decoding algorithm. Two kinds of outputs can be set for the MLPNN. One way is to use the 10 category labels as the outputs, similar with the PNN output. The other way is to use the concentrations of the two pheromones as the output targets and then calculate the ratio to determine the category. More output nodes in the former method will produce a larger neural network to train, as the number of hidden layer nodes is generally set to more than the number of output nodes to utilize the generality of NNs. This would greatly increase the training time. Therefore, the latter approach was chosen here.

Ten category output targets are defined as [1.00, 0], [0.66, 0.33], [0.50, 0.50], [0.33, 0.66], [0, 1.00], [2.00, 0], [1.33, 0.66], [1.00, 1.00], [0.66, 1.33], [0, 2.00]. The designed MLPNN structure is shown in Figure 5b. A sigmoid function is used as the active function in the hidden layer neurons. A linear function is used in the output layer. The output values are classified into 10 categories based on the distances to the possible targets.

## 6. Results and Analyses

The extracted sensor features in Section 4 are further processed by the LDA, PNN, and MLPNN classification techniques, as described in Section 5. The recognition rate and speed are determined, the performance of recognition based on single features, combined features, and sequential selected features are calculated.

### 6.1. Decoding Based on Single Feature

**Figure 6:**
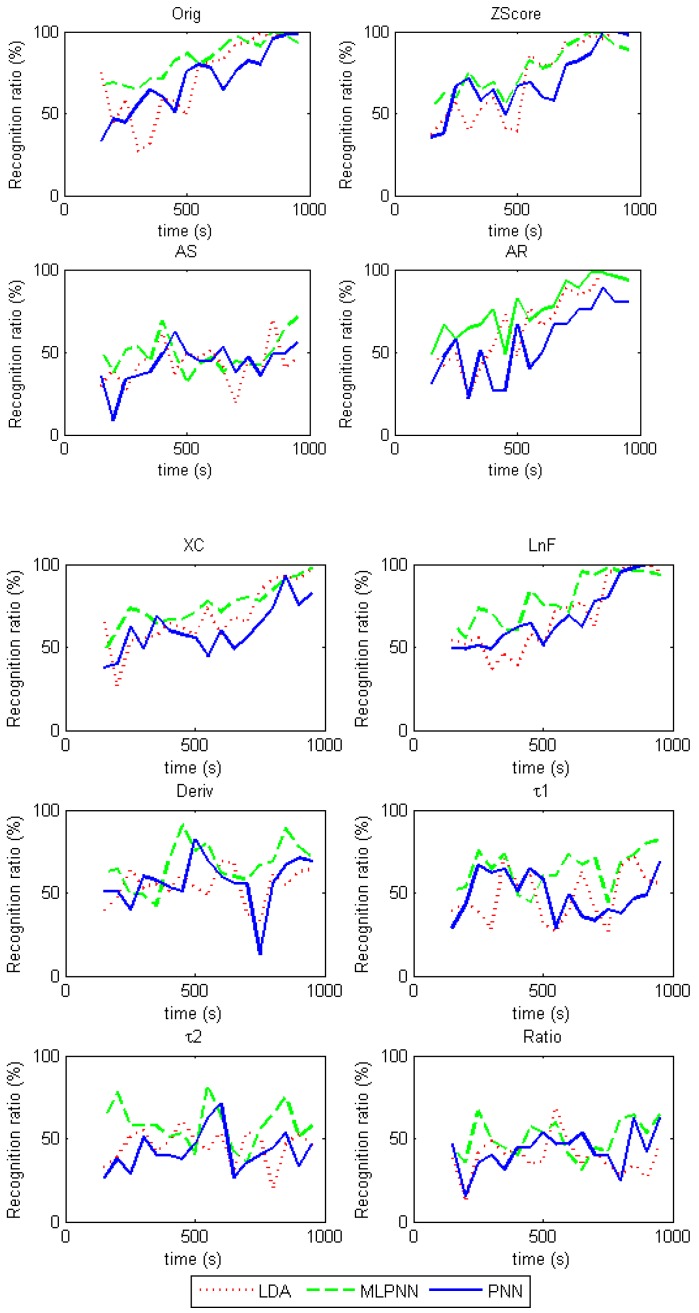
Classification comparison based on three methods using 45 measurements.

Each extracted feature in Table 2 is used as the input of the LDA, PNN, and MLPNN recognition algorithms. The Leave-One-Out verification method is adopted and the average recognition rates based on the features are shown in Figure 6.

It was observed that the *AS* and *Ratio* features were not effective for recognition, as the recognition ratio was roughly lower than 50% for each. For features based on the steady state responses, including *Orig*, *Zscore*, *AR*, *XC* and *LnF*, recognition rates are approximately 50% in the initial period (200 s–500 s), and improve with time, which could reach over 90% after 800 s. Among these parameters, *XC* was the worst from the view of recognition ratio and time to reach the highest score. For features based on the dynamic responses, including *Deriv*, τ1 and τ2, recognition rates are roughly 70% in the period of 300 s–700 s, and they remain similar over time.

For the LDA, PNN, and MLPNN recognition results shown in Figure 6, MLPNN shows slightly better recognition ratio but required much longer training times.

### 6.2. Decoding Based on Feature Combination

Recognition rates using steady state features increase over time but are low in the beginning as shown in Figure 6. However, recognition rates using the transient features are higher at the beginning. A combination of features has the potential to improve the overall recognition rate.

**Figure 7:**
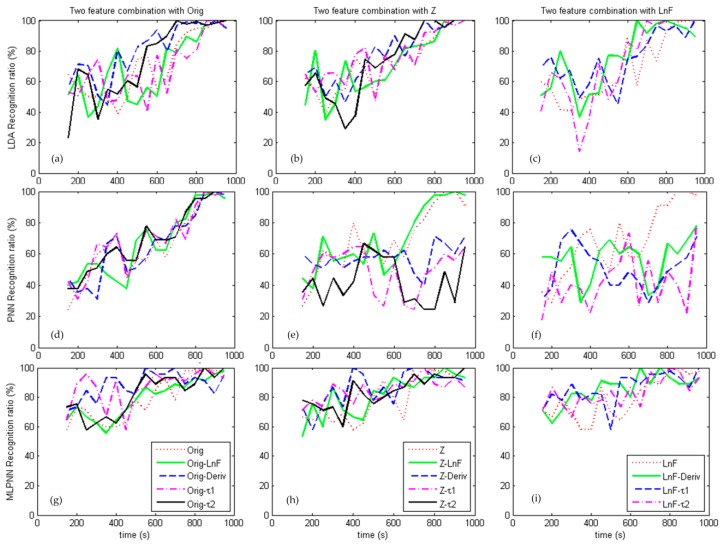
Linear Discriminant Analysis (LDA), Probabilistic Neural Network (PNN), and Multilayer Perception Neural Network (MLPNN) recognition based on combination of feature. From left to right, LDA recognition ratio based on two feature combinations with (**a**) *Orig*; (**b**) *Z*; (**c**) *LnF*, PNN recognition ratio based on two feature combinations with (**d**) *Orig*; (**e**) *Z*; (**f**) *LnF*, and MLPNN recognition ratio based on two feature combinations with (**g**) *Orig;* (**h**) *Z;* and (**i**) *LnF*.

Combinations of features are also fed into the LDA, PNN, and MLPNN algorithms, and the results are shown in Figure 7. The used features are *Orig*, *Zscore*, *LnF*, *Deriv*, τ1, and τ2.

Figure 7 shows the recognition rate of LDA, PNN, and MLPNN using the combinations of steady features and transient features. Figure 7a shows the LDA recognition rate of combination of the *Orig* feature with others and the *Orig-*τ2 combination shows the fastest recognition rate, reaching 100% ratio at 700 s. Feature combination does improve recognition compared with Figure 6, especially for combinations with dynamic features. Figure 7b shows the combination of the standardized score with others and the *Z-*τ2 combination reaches 100% ratio at 750 s. Figure 7c shows the combination of the *LnF* with others and the *LnF-Deriv* combination achieved 100% ratio at 650 s.

The PNN recognition rate based on feature combinations are shown in the second row of Figure 7. A combination of *Orig* with other features shows improved results in Figure 7d and the performances are similar reaching over 90% ratio at 800 s. Figure 7e shows that the *Z*-*LnF* combination gets 97% ratio at 800 s, while the other combinations show no improvement. Combinations of *LnF* with other features shows no improvement, says in Figure 7f.

Figure 7g–i (third row) shows the recognition rate of MLPNN using the combined features. The improvement of feature combination can be seen clearly and roughly the MLPNN shows better performance than LDA and PNN. *Orig-*τ2, *Z-*τ2, and *LnF-Deriv* reach 100% ratio at 550 s, 400 s and 650 s, respectively.

In summary, combining a steady state feature and a transient feature can improve the recognition rate for LDA and MLPNN algorithms, but less so for the PNN algorithm.

### 6.3. Decoding Based on Sequential Forward Feature Selection

Feature subset selection (FSS) is an important feature selection technique that can be used to find an optimal subset of features that maximizes information content or predictive accuracy [24]. Sequential search algorithms are computer-intensive strategies that reduce the number of states to be visited during search by applying local search. The simplest methods are sequential forward selection (SFS) and sequential backward selection (SBS) [31]. In this work, the SFS approach has been adopted for the sake of expediency.

The input feature set is designed as {*Orig*, *Zscore*, *AR*, AS, *LnF*, *Deriv*, τ1, τ2}. The recognition rate with SFS based PNN and MLPNN classification algorithms are calculated and the features selected are shown in Table 3. Compared to Figure 7, there is no clear improvement of the recognition rate. Again, it can be seen that transient features play greater roles than steady state features during earlier stage to improve the recognition rate, while steady features play higher roles lately. Among the steady features, *Orig* and *LnF* show up more than others. *Deriv* shows more than others in transient features. PNN obtains better recognition rate and faster computation than MLPNN.

### 6.4. Expanding Input Samples with Neighbours

The challenge of this ratiometric decoding problem is the limitation of pre-tested sensor responses. This confined data-set has restricted the recognition rate and recognition speed. However, as the data acquisition interval is far less than the sensor response time, the closest data-points in time also are closest in values. Therefore, nearest neighbour is adopted as the input of recognition algorithms. By utilizing this approach, the whole sample set is expanded to 90. Together with the SFS based recognition methods, greatly improved results are obtained. The LDA based approach can be used together with SFS after expanding the data set, as it overcomes the limitation described above. But for MLPNN, the expanding of dataset brings an extremely long training period. Therefore, only the LDA and PNN are applied in this section.

**Figure 8:**
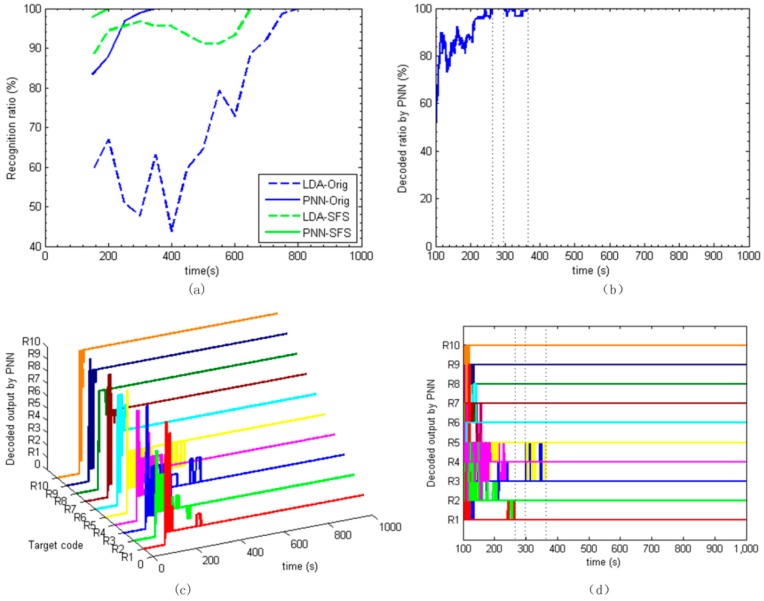
(**a**) LDA and PNN recognition performance using the extended dataset based on *Orig* and SFS feature set; (**b**) the real-time decoding ratio of PNN; (**c**) decoded trajectory plot of PNN; and (**d**) two-dimensional (2D) decoded output by PNN.

The recognition results of LDA and PNN using the expanded data-set are shown in Figure 8a. The calculation time step is 50 s. It can be seen that when using the single feature of *Orig* in the expanded data set, the recognition ratio of PNN reaches 100% at 350 s, showing a greatly improved performance, while LDA recognition performance is slightly improved when compared with Figure 7a. However, the SFS based LDA and PNN achieve excellent performances. The SFS based LDA approach obtains more than 90% recognition ratio at 200 s, producing significant improvement. The recognition ratio of 100% is achieved at 200 s by the SFS based PNN, which means the target information is decoded 100 s after the injection of the pheromone blend.

### 6.5. Real-Time Decoding

Based on the above analysis, when considering the recognition rate and the amount of computation, the PNN based on the expanded data-set is preferred as the final real-time decoding approach. Information at each time is recognized by PNN.

To verify the decoding approach, the sensor frequency shifts are processed with the time interval of 1 s. Figure 8c shows the real-time decoded results plotted 3D over time. The decoded outputs are 0 at the beginning 100 s as there are no pheromone blends. When the pheromone blends are injected at 100 s, outputs show up as the 10 codes of from R1 to R10. The recognized codes jump between similar and most adjacent response categories. Initially, R1 has been recognized as R1, R10, and R9 at the beginning, then jumps to R2 and finally goes to the target code of R1 at about 150 s. Roughly, R1, R2, R4, R6, R7, R8, R9, and R10 are decoded out before 200 s.

The total recognition ratio is illustrated in Figure 8b. The recognition ratio achieves 100% around 270 s and slightly down to 90% during the period of 300 s to 360 s, because R3 and R5 mixed together, as shown in Figure 8d. After 360 s, the recognition ratio stays at 100% till the end. Hence, we can say that all of the settled chemical information is decoded out roughly after 360 s. This decoding time is greatly shorter than the other approaches. The decoding process is clear, effective, and fast.

## 7. Conclusions

A biomimetic infochemical communication system in which insect pheromones are employed as molecular messengers is presented and infochemical transferring experiments have been performed. Information has been ratiometrically encoded using binary mixtures of two kinds of pheromones with different concentrations, and then released into a small wind tunnel mimicking the infochemical transfer process. These uniquely encoded chemical signals were successfully detected by an array of polymer coated SAWR sensors and ratiometric classifier. Different ratiometric decoding approaches have been studied and an effective real-time decoding method is described for our infochemical communication system.

Because sensor responses show low stability and high correlations, it is difficult to classify the data points by PCA with the original signal. Eight features have been extracted out and utilized in analyzing the SAWR sensor responses. LDA, PNN, and MLPNN algorithms have been used to analyze different sets of sensor features. *Orig*, *Zscore*, *LnF*, *Deriv*, τ1, τ2 are useful features and selected as a good feature subset, including steady state and transient values. Results show that the combination of steady state and transient features improve the LDA and MLPNN recognition performances. A sequential forward feature selection method was combined with the three algorithms to improve optimal feature selection. Important selected features are *Orig* and *LnF*. *Orig* worked best for PNN, and *LnF* for MLPNN. MLPNN shows improved performance based on combinations of steady and transient features with large computation consumption. 

Significant improvement was achieved by using the adjacent neighbouring data-points to expand the data-set. The SFS based PNN obtains 100% ratio at 200 s with a time interval of 50 s. SFS is time consuming. Final real-time decoding is performed by PNN using only *Orig* feature and a greatly improved recognition performance is achieved. Decoding ratio of 100% is obtained at about 260 s after the injection of chemical blends, while maximum sensor response occurs at about 600 s after injection.

In conclusion, the system described in this paper has been shown to classify successfully all of the ratiometric information, thus mimicking a complete insect-based infochemical communication system. The sensor response time, and consequently decoding rate, could be improved by reducing spatial scales utilizing nano-engineered VLSI systems, thereby improving temporal precision. Such a biomimetic system could serve as the foundation for a new form of low-cost information transmission for broadband chemical communication influencing a range of applications, such as environmental monitoring, product labeling, medical diagnosis, and nanoscale communication.

## Figures and Tables

**Table 1:** Ratios of the two pheromones used to demonstrate the basic principle of ratiometric infochemical communication.

Ratios	1:0		2:1		1:1		1:2		0:1	
Categories	R1	R6	R2	R7	R3	R8	R4	R9	R5	R10
E11-14:OAc volume (μL)	1	2	0.66	1.33	0.5	1	0.33	0.66	0	0
Z9-14:OAc volume (μL)	0	0	0.33	0.66	0.5	1	0.66	1.33	1	2

**Table 2:** List of extracted features.

Description	Designator	Definition ^1^
Original signal	*Orig*	$f_{ij}\left(t\right)$
Standardized score	*Zscore*	$z_{ij}\left(t\right)=[f_{ij}\left(t\right)\;-\;\mu_{j}\left(t\right)]/\sigma_{j}\left(t\right)$
Autoscaled value	*AS*	$AS_{ij}\left(t\right)\;=f_{ij}\left(t\right)/L_{i}\left(t\right)$, where $L_{i}\left(t\right)\;={\left[\overset{3}{\underset{j=1}{\sum }}{\left(f_{ij}\left(t\right)\;\right)}^{2}\right]}^{1/2}$
AutoRanged value	*AR*	$AR_{ij}\left(t\right)\;=f_{ij}\left(t\right)/R_{j}\left(t\right),\;R_{j}\left(t\right)\;={\left[\frac{1}{N}\overset{N}{\underset{i=1}{\sum }}{\left(f_{ij}\left(t\right)\;\right)}^{2}\right]}^{1/2}$
Simple scaling by concentration ratio	*XC*	$XC_{ij}\left(t\right)\;=f_{ij}\left(t\right)/C_{i},C_{i}=\{\begin{array}{c} 1,\;i=1,2,3,\;\ldots ,30\\ 2,\;i=31,32,\ldots ,45\; \end{array}$
Logarithm of frequency	*LnF*	$LnF_{ij}\left(t\right)\;=\ln\left(\left|f_{ij}\left(t\right)\;\right|\right)$
1st order derivative of frequency	*Deriv*	$DF_{ij}\left(t\right)=df_{ij}\left(t\right)/dt$
The time constant 1	*τ1*	$\tau 1_{ij}\left(t\right)\;=-1/\left\{d\left\{\ln\left[f_{ij}\left(\infty \right)-f_{ij}\left(t\right)\right]\right\}/dt\right\}$
The time constant 2	*τ2*	$\tau 2_{ij}\left(t\right)\;=1/\left[\frac{df_{ij}\left(t\right)}{dt}\frac{1}{f_{ij}\left(\infty \right)}\right]=f_{ij}\left(\infty \right)/\left[\frac{df_{ij}\left(t\right)}{dt}\right]$
Ratio between sensors	*RatioF*	$R_{ij}\left(t\right)=\left[\frac{f_{i1}\left(t\right)}{f_{i2}\left(t\right)},\frac{f_{i1}\left(t\right)}{f_{i3}\left(t\right)},\frac{f_{i2}\left(t\right)}{f_{i3}\left(t\right)}\right]$

^1^ Subscript i denotes sample number, subscript j denotes sensor number.

**Table 3:** Table detailing the features selected at time stations with their corresponding recognition rates obtained using sequential forward selection (SFS) based PNN and MLPNN algorithms.

Time(s)	PNN		MLPNN	
	Features	Recognition Rate	Features	Recognition Rate
150	*Deriv*	51.1%	*LnF*	35.6%
200	*Deriv*, *τ*1	57.8%	*LnF*, *τ*1, *τ*2	60.0%
250	*Deriv*, *τ*1, *τ*2	64.4%	*Deriv*, *τ*1, *τ*2	57.8%
300	*Zscore*, *τ*1	64.4%	*Orig*, *LnF*	57.8%
350	*Orig*	62.2%	*LnF*, *τ*2	64.4%
400	*Orig*	64.4%	*Orig*, *Deriv*	68.9%
450	*Deriv*	62.2%	*LnF*, *Deriv*	73.3%
500	*Zscore*, *Deriv*	68.9%	*Deriv*	60.0%
550	*Zscore*	64.4%	*Orig*	62.2%
600	*Orig*	66.7%	*AS*	73.3%
650	*Orig*	66.7%	*LnF*	75.6%
700	*Orig*	75.6%	*Orig*, *AS*, *LnF*, *Deriv*	82.2%
750	*Zscore*	86.7%	*LnF*, *Deriv*	84.4%
800	*LnF*	93.3%	*Orig*, *AS*	88.9%
850	*Orig*, *τ*1	100.0%	*LnF*	84.4%
900	*Orig*	97.8%	*LnF*	84.4%
950	*Orig*	97.8%	*AS*	80.0%

## References

[1] Ngo T.D. (2015). Biomimetic Technologies: Principles and Applications.

[2] Hansson B.S., Stensmyr M.C. (2011). Evolution of insect olfaction. Neuron.

[3] Shen K., Tootoonian S., Laurent G. (2013). Encoding of mixtures in a simple olfactory system. Neuron.

[4] Rácz Z., Cole M., Gardner J.W., Chowdhury M.F., Bula W.P., Gardeniers J.G.E., Karout S., Capurro A., Pearce T.C. (2013). Design and umplementation of a modular biomimetic infochemical communication system. Int. J. Circuit Theory Appl..

[5] Sakurai T., Mitsuno H., Haupt S.S., Uchino K., Yokohari F., Nishioka T., Kobayashi I., Sezutsu H., Tamura T., Kanzaki R. (2011). A single sex pheromone receptor determines chemical response specificity of sexual behavior in the silkmoth Bombyx mori. PLoS Genet..

[6] Löfstedt C. (1990). Population variation and genetic control of pheromone communication systems in moths. Entomol. Exp. Appl..

[7] Kuebler L.S., Olsson S.B., Hansson B.S. (2011). First order processing of complex olfactory information in the moth brain. Procedia Computer Science.

[8] Olsson S.B., Challiss R.A.J., Cole M., Gardeniers J.G.E., Gardner J.W., Guerrero A., Hansson B.S., Pearce T.C. (2015). Biosynthetic infochemical communication. Bioinspir. Biomim..

[9] Malo E.A., Renou M., Guerrero A. (2000). Analytical studies of spodoptera littoralis sex pheromone components by electroantennography and coupled gas chromatography—Electroantennographic detection. Talanta.

[10] Ljungberg H., Anderson P., Hansson B.S. (1993). Physiology and morphology of pheromone-specific sensilla on the antennae of male and female *Spodoptera littoralis* (Lepidoptera, Noctuidae). J. Insect Physiol..

[11] Yang J., Rácz Z., Gardner J.W., Cole M., Chen H. (2012). Ratiometric info-chemical communication system based on polymer-coated surface acoustic wave microsensors. Sens. Actuators B Chem..

[12] Bula W.P., Dimov N.G., Guerrero A. Artificial gland for precise release of semiochemicals for chemical communication. Proceedings of the 14th International Conference on Miniaturized Systems for Chemsity Life Science.

[13] Cole M., Rácz Z., Gardner J.W., Pearce T.C. A novel biomimetic infochemical communication technology: From insects to robots. Proceedings of the IEEE Sensors Conference.

[14] Thomas S., Leong S.L.T., Rácz Z., Cole M., Gardner J.W. Design and implementation of a high-frequency surface acoustic wave sensor array for pheromone detection in an insect-inspired infochemical communication system. Proceedings of the 4th International Conference on Miniaturized Systems for Chemistry and Life Sciences.

[15] Rácz Z., Olsson S.B., Gardner J.W., Pearce T.C., Hansson B.S., Cole M. (2011). Challenges of biomimetic infochemical communication. Procedia Computer Science.

[16] Rácz Z., Gardner J.W., Cole M. Volatile-based ratiometric infochemical communication system using polymer-coated piezoelectric sensor arrays. Proceedings of the IEEE Sensors Conference.

[17] Thomas S., Villa-Lopez F., Theunis J., Peters J., Cole M., Gardner J. (2015). Particle System using Solidly Mounted Resonators. IEEE Sens. J..

[18] Muñoz L., Rosell G., Quero C., Guerrero A. (2008). Biosynthetic pathways of the pheromone of the Egyptian armyworm Spodoptera littoralis. Physiol. Entomol..

[19] Dimov N., Muñoz L., Carot-Sans G., Verhoeven M.L.P.M., Bula W.P., Kocer G., Guerrero A., Gardeniers H.J.G.E. (2011). Pheromone synthesis in a biomicroreactor coated with anti-adsorption polyelectrolyte multilayer. Biomicrofluidics.

[20] Marco S., Gutiérrez-gálvez A. (2012). Signal and Data Processing for Machine Olfaction and Chemical Sensing: A Review. IEEE Sens. J..

[21] Yan S., Spangler W.S., Chen Y. (2013). Chemical Name Extraction Based on Automatic Training Data Generation and Rich Feature Set. IEEE/ACM Trans. Comput. Biol. Bioinform..

[22] Pardo M., Sberveglieri G. (2004). Remarks on the Use of Multilayer Perceptrons for the Analysis of Chemical Sensor Array Data. IEEE Sens. J..

[23] Tian G.Y., Sophian A., Taylor D., Rudlin J. (2005). Wavelet-based PCA defect classification and quantification for pulsed eddy current NDT. IEEE Proc. Sci. Meas. Technol..

[24] Gutierrez-Osuna R. (2002). Pattern analysis for machine olfaction: A review. IEEE Sens. J..

[25] Krzanowski W.J. (1988). Principles of Multivariate Analysis: A User’s Perspective.

[26] Bur C., Andersson M., Spetz A.L., Helwig N., Schutze A. (2014). Detecting Volatile Organic Compounds in the ppb range with platinum-gate SiC-Field Effect Transistors. IEEE Sens. J..

[27] Kohl C.-D., Wagner T. (2014). Gas Sensing Fundamentals.

[28] Wasserman P.D. (1993). Advanced Methods in Neural Computing.

[29] Jatmiko W., Fukuda T., Sekiyama K. Optimized probabilistic neural networks in recognizing fragrance mixtures using higher number of sensors. Proceedings of the 2005 IEEE Sensors.

[30] Zheng X.Z.X., Jia J.J.J., Hao Y.H.Y., Zhang S.Z.S., Chen W.C.W., Dai J.D.J. Probabilistic neural network based motor cortical decoding method and hardware implementation. Proceedings of the 2010 IEEE International Symposium on Computer-Aided Control System Design.

[31] Devijver P.A., Kittler J. (1982). Pattern Recognition, A Statistical Approach.

